# Estimating correlations across tasks in experimental psychology

**DOI:** 10.3758/s13428-026-02990-6

**Published:** 2026-03-30

**Authors:** Shanglin Yang, Jeffrey N. Rouder

**Affiliations:** Department of Cognitive Science, University of California, Irvine, CA 92697 USA

**Keywords:** Individual differences, Correlations, Hierarchical models, Prior selection, Bayesian analysis

## Abstract

Understanding how people covary in performance across experimental tasks is central to individual-difference psychology. The classic Pearson correlation has two strengths: (1) it is invariant to the scale of measurement, and (2) it is invariant to including additional variables in the analysis. However, it is susceptible to attenuation from measurement noise. Bayesian hierarchical models address this issue by modeling measurement error directly. Resulting estimates, however, depend on prior specifications and are not invariant to scale or variable inclusion. We compare three common priors—inverse Wishart (IW), scaled inverse Wishart (SIW), and LKJ—to assess robustness to prior assumptions in hierarchical settings. Our main tools are visualizing the priors and evaluating their effects on posterior estimates through simulation. When prior settings match ground truth, all priors recover true correlations accurately in low-dimensional settings. When prior variance is misspecified, the IW shows strong bias: low-variance priors inflate correlations, and high-variance priors deflate them. The SIW shows the same pattern but less severely, while the LKJ remains largely unaffected by scale misspecification. When more variables are added, the IW is most stable, whereas the SIW and LKJ show slight shrinkage toward lower correlations. The main drawback of the LKJ is computational speed—models with it can take orders of magnitude longer than those using IW or SIW. Overall, the LKJ provides the most accurate estimates, while the SIW offers a practical compromise for large-scale models where computational speed is crucial.

It is common in experimental psychology to study how people covary across tasks. An example is assessing whether people who are highly susceptible to Stroop interference are also susceptible to Flanker interference. Researchers who study individual differences hope that the pattern of observed correlations may provide insight into the nature and dimension of processing. Here are two well-known examples where this hope was realized: First, in personality psychology, the pattern of correlations across various personality questionnaire items forms the evidence for the Big 5 theory of personality (McCrae & Costa, 1997). Second, in cognitive psychology, the pattern of correlations across executive-function tasks forms the evidence for various theories of executive function, including the inhibition–shifting–updating theory of Miyake et al. (2000). Although each of these theories has been criticized, the general method of studying covariation across tasks remains timely and topical.

**Fig. 1:**
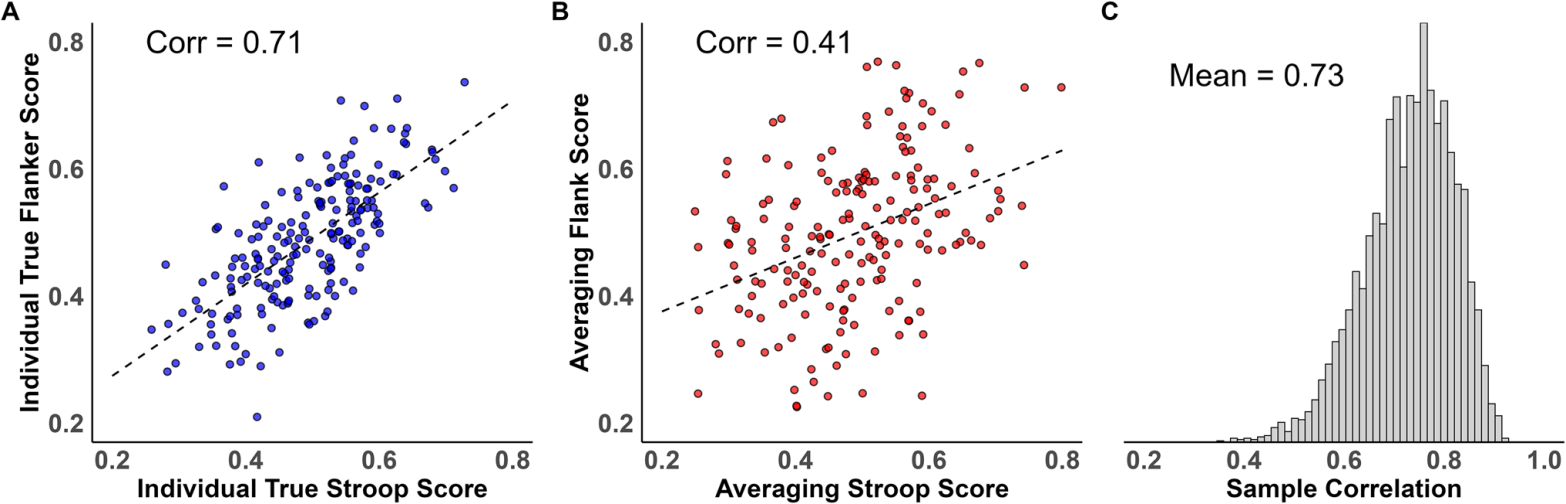
The effects of trial variability on correlation estimates. Synthetic individuals were sampled from a bivariate distribution with a true population correlation of .7. **A** Scatter plot among individual true values for 200 individuals. The correlation is .73 in value, which is close to the true value. **B** True values from individuals are not known and must be estimated from trial data by averaging. If the trials are too few or excessively noisy, the averaged individuals’ scores are themselves randomly perturbed from their true values. This perturbation systematically attenuates the observed correlation among the tasks. **C** Hierarchical models provide separate estimates of trial noise and covariance across individuals in tasks. By separately modeling different sources of variation, the posterior estimates of correlation are disattenuated; moreover, uncertainty in the posterior reflects limitations from the finite samples of participants and trials

Estimating correlations at first glance seems straightforward: Point estimates come from Pearson’s sample correlation formula (Pearson, 1900); confidence intervals (CIs) come from Fisher’s *z*-transform method (Fisher, 1921). Each of these computations has the following beneficial property: The analyst can change the *location* and *scale* of the variables without changing the estimation of correlation. For example, suppose we are studying the association between heart rate and ambient temperature. One analyst chooses to measure temperature in Celsius while another in Fahrenheit, even though the measures vary in location (where the zero point is) and scale (how big a degree is). Fortunately, the point estimate and associated CIs do not depend on this choice—the same values are obtained under a linear transformation of either variable. This property may be termed *location and scale invariance*. Even more impressively, each of the computations displays *invariance to inclusion of other variables*. For example, suppose one researcher considered height and weight in isolation and another considered height, weight, shoe size, and waist circumference simultaneously. It is not obvious that the correlation coefficient between weight and height would be the same for both researchers. In the latter case, the resulting set of correlations in the correlation matrix must be positive semidefinite, implying a common constraint. Yet, it may be shown that the correlation for the latter depends only on height and weight, and the point estimate and CIs are the same in both cases. Given these two advantages, as well as the ease of computation, estimation of correlations seems a solved problem.

A critical complication comes when variables are measured with substantial measurement error, as they almost always are in psychology. Let’s take the following case where each of many individuals participates in two psychological tasks. Let $Y_{i1}$ and $Y_{i2}$ be the scores for the $i$th individual, $i=1,\ldots ,I$ for Tasks 1 and 2, respectively. To incorporate measurement error, let

$$ Y_{ij} \mid \theta _{ij} \sim \text{ N }(\theta _{ij},\tau ^2_j), \quad j=1,2, $$

where $\theta _{i1}$ and $\theta _{i2}$ are true scores for the $i$th individual for Tasks 1 and Task 2, respectively. The terms $\tau _1$ and $\tau _2$ are the error variance for the two tasks. Figure 1A shows a scatter plot of hypothetical individual true scores $\theta _{i1}$ and $\theta _{i2}$ for $I=200$ individuals. These are correlated; the true population value is .7 and the goal is to recover this value. The sample correlation for these 200 individuals is .71, which is quite close to the true population value. Figure 1B shows the scatter plot of observed scores $Y_{i1}$ and $Y_{i2}$, and again, we wish to recover the true value of .7. These observed scores are perturbed by error in random directions and to random degrees. The net result is that the correlation is attenuated (Spearman, 1904). The sample correlation on observed scores is .41 in value, which is quite far from the true population value of .7.

More formal development is instructive in addressing the problem. First, a bivariate normal is placed on true scores:

$$\hat{A} \begin{pmatrix} \theta _{i1}\\ \theta _{i2} \end{pmatrix} \sim \text{ N}_2\left( \begin{pmatrix} \mu _1 \\ \mu _{2} \end{pmatrix}, \begin{pmatrix} \sigma ^{2}_{1} & \rho \sigma _{1}\sigma _{2}\\ \rho \sigma _{1}\sigma _{2} & \sigma ^{2}_{2} \end{pmatrix}\right) . $$

The key quantity here is population correlation $\rho$, and it is this quantity we hope to recover. Of course, we do not observe true scores, but the observations. The distribution on $Y_{ij}$ is:

$$ \begin{pmatrix} Y_{i1}\\ Y_{i2} \end{pmatrix} \sim \text{ N}_2\left( \begin{pmatrix} \mu _1 \\ \mu _2 \end{pmatrix}, \begin{pmatrix} \sigma ^2_1 +\tau _1^2& \rho \sigma _1\sigma _2\\ \rho \sigma _1\sigma _2 & \sigma ^2_2 + \tau _2^2 \end{pmatrix}\right) . $$

From this distribution, it is now clear that the sample correlation from observed scores, denoted $\hat{\rho }$ measures

$$ E(\hat{\rho }) \approx \left( \frac{\sigma _1\sigma _2}{\sqrt{\sigma _1^2+\tau _1^2} \sqrt{\sigma _2^2+\tau _2^2}}\right) \rho . $$

The coefficient here describes attenuation as the denominator is necessarily larger than the numerator. And without any further information about measurement noise, we are unsure by how much.

Our application is to experimental psychology, and tasks tend to be comprised of many repeated trials. The usual course of analysis is to average across replicates to form individual-by-task scores, which are then correlated. There is noise in these trials, which means the averages also have noise, although certainly less so. This trial noise affects the average attenuation results. Moreover, in this averaging approach, the only way to reduce attenuation is to increase the number of trials per individual. It cannot be reduced by adding more individuals, as adding individuals simply adds more perturbed points to the scatter plot.

An attractive alternative to averaging for disattenuating correlations in analysis is through hierarchical models (Haines et al., 2025; Matzke et al., 2017; Rouder & Haaf, 2019). In a hierarchical model, multiple sources of noise are specified and all the data, not just the averages, are used. For the above example, trial noise as well as covariability of people in tasks are included. Hierarchical models have become popular in experimental psychology because they lead to more accurate estimation and inference across a wealth of paradigms (Rouder & Lu, 2005; Rouder & Province, 2019). In this case, the separation of trial noise from variability across tasks in suitable hierarchical models leads to disattenuated estimates of correlations (Rouder et al., 2023; Rouder & Mehrvarz, 2024). As proof of concept, Figure 1C shows the posterior distribution of $\rho$ when all the data are analyzed in a subsequently presented hierarchical model. As can be seen, they are centered close to the true value of .7 rather than the attenuated value of .41.

Estimation of correlation in hierarchical models is not without difficulties. The measurement invariances in sample correlations—invariance to location and scale and invariance to inclusion of additional variables—may not be attainable or even desirable in hierarchical settings.

Analysis of hierarchical models is convenient in the Bayesian framework (Gelman et al., 2004). This paper addresses how to estimate a correlation matrix in both hierarchical and conventional normal models. A key issue is the specification of the prior. How does the choice of prior affect the posterior distribution of correlation coefficients? We frame our assessment in terms of the measurement invariances highlighted above. Invariance to location occurs fairly naturally for reasons discussed subsequently and will not play a role. Invariance to scale and invariance to the inclusion of additional variables are more difficult with the methods we present. For example, in all the prior specifications discussed here, the analyst must set a scale or expectation of the degree of variability beforehand. Robustness to scale specification occurs when the posterior of correlation coefficients is relatively unaffected by reasonable variation in this scale setting. Likewise, robustness to inclusion occurs when the posterior of correlation coefficients is relatively unaffected by the inclusion or exclusion of a handful of additional variables.

We study the following prior classes for correlations: The inverse Wishart prior (O’Hagan & Forster, 2004), which offers advantages of conjugacy, computational convenience; the scaled inverse Wishart prior (Huang & Wand, 2013), which is a continuous mixture of inverse Wishart components and provides more flexibility in modeling variances; the LKJ prior (Lewandowski et al., 2009), which is less informative than either Wishart-based alternatives (Tokuda et al., 2025). This paper explores the conditions under which each of these choices is useful for estimating correlations. Our main approach to comparing these models is through simulation with known truths. In each simulation run, posterior distributions of population-level correlations are compared to these truths. This simulation approach is precedented for covariance matrices, and examples include Rouder et al. (2007), Schuurman et al. (2016); Liu et al., 2016. To our knowledge, however, we are the first to do so for assessing correlations across tasks in experimental designs. Moreover, although the inverse Wishart prior has been studied extensively, there is far less work with the scaled inverse Wishart and LKJ priors, perhaps because they are relatively new and only incorporated into Jags and Stan in the last decade.

## Manifest and hierarchical models

We use the term *manifest* model for the conventional case where researchers place a multivariate model on observables, and manifest models are appropriate when there is an ignorable degree of measurement error. Examples of variables that we may treat as free of measurement error are body metrics such as height, weight, and waist circumference. This case is easy in the conventional setup; one simply uses sample correlations along with Fisher confidence intervals. For the Bayesian case, the manifest model is given as follows: Let $Y_{ij}$ denote the score for the $i$th individual, $i=1,\ldots ,I$ on the $j$th task, $j=1,\ldots ,J$, and let $\boldsymbol{Y}_i=(Y_{i1},\ldots ,Y_{iJ})'$ be a column vector of scores for the $i$th individual:

$$ \text{ Manifest } \text{ Model }: \quad \quad \boldsymbol{Y}_i \sim \mathrm{ N}_J(\boldsymbol{\mu },\boldsymbol{\Sigma }), $$

where $\boldsymbol{\mu }=(\mu _1,\ldots ,\mu _J)'$ is a column vector of means per task and $\boldsymbol{\Sigma }$ is a variance matrix:

$$ \boldsymbol{\Sigma } = \begin{bmatrix} \sigma ^2_1 & \rho _{12} \sigma _1\sigma _2 & \ldots & \rho _{1J}\sigma _1\sigma _J\\ \rho _{12} \sigma _1\sigma _2 & \sigma ^2_2 & \ldots & \rho _{2J}\sigma _2\sigma _J\\ \vdots & \vdots & \ddots & \vdots \\ \rho _{1J} \sigma _1\sigma _J & \rho _{2J}\sigma _2\sigma _J & \ldots & \sigma _J^2 \end{bmatrix}. $$

The covariance matrix can be expressed in terms of a correlation matrix as follows:

$$ \boldsymbol{\Sigma }= \begin{bmatrix} \sigma _1 & 0 & \ldots & 0\\ 0 & \sigma _2 & \ldots & 0 \\ \vdots & \vdots & \ddots & \vdots \\ 0 & 0 & \ldots & \sigma _J \end{bmatrix} \times \begin{bmatrix} 1 & \rho _{12} & \ldots & \rho _{1J}\\ \rho _{12} & 1& \ldots & \rho _{2J} \\ \vdots & \vdots & \ddots & \vdots \\ \rho _{1J} & \rho _{2J} & \ldots & 1 \end{bmatrix} \times \begin{bmatrix} \sigma _1 & 0 & \ldots & 0\\ 0 & \sigma _2 & \ldots & 0 \\ \vdots & \vdots & \ddots & \vdots \\ 0 & 0 & \ldots & \sigma _J \end{bmatrix}, $$

which may be written compactly as:

1 $$\begin{aligned} \boldsymbol{\Sigma } = D(\boldsymbol{\sigma }) \boldsymbol{\rho } D(\boldsymbol{\sigma }), \end{aligned}$$

where $D(\boldsymbol{\sigma })$ is a diagonal matrix with $\boldsymbol{\Sigma }=(\sigma _1,\ldots ,\sigma _J)$ on the diagonal and $\boldsymbol{\rho }$ is the correlation matrix. Priors on $\boldsymbol{\mu }$ may be set broadly, making the model robust to changes in location.

A hierarchical model is appropriate when there are several repeated observations for each individual in each task. Each individual performs $L_{ij}$ trials on each task, and the score on each trial is denoted $Y_{ij\ell }$, where, as before $i$ indexes individual, $i=1,\ldots ,I$, $j$ indexes task, $j=1,\ldots ,J$, and $\ell$ indexes replicate, $\ell =1,\ldots ,L_{ij}$. The simplest model is

$$ \begin{aligned} \text{ Hierarchical } \text{ Model: } \quad \quad&Y_{ij\ell } \mid \theta _{ij} \sim \text{ N }(\theta _{ij},\tau _j^2),\\&\boldsymbol{\theta }_{i} \sim \mathrm{ N }(\boldsymbol{\mu },\boldsymbol{\Sigma }). \end{aligned} $$

The above hierarchical model consists of two levels. The first a *data-level* specification that describes how scores on trials vary with people and tasks. Parameter $\theta _{ij}$ is the true score for the $i$th person in the $j$th task. The term $\tau ^2_j$ describes the trial-to-trial variability for the $j$th task. Although it is possible to expand this model to include conditions, covariates, and other distributional families, this simplified model is ideal for exploring the effects of priors in Bayesian analysis. The second level is the *individual-level* specification that describes how individual’s performance covary across tasks. An individual’s profile $\boldsymbol{\theta }_i=(\theta _{i1},\ldots ,\theta _{iJ})'$ is a column vector of true scores, and the correlation among tasks, the target of study, is contained within $\boldsymbol{\Sigma }$ via Equation (1).

**Fig. 2:**
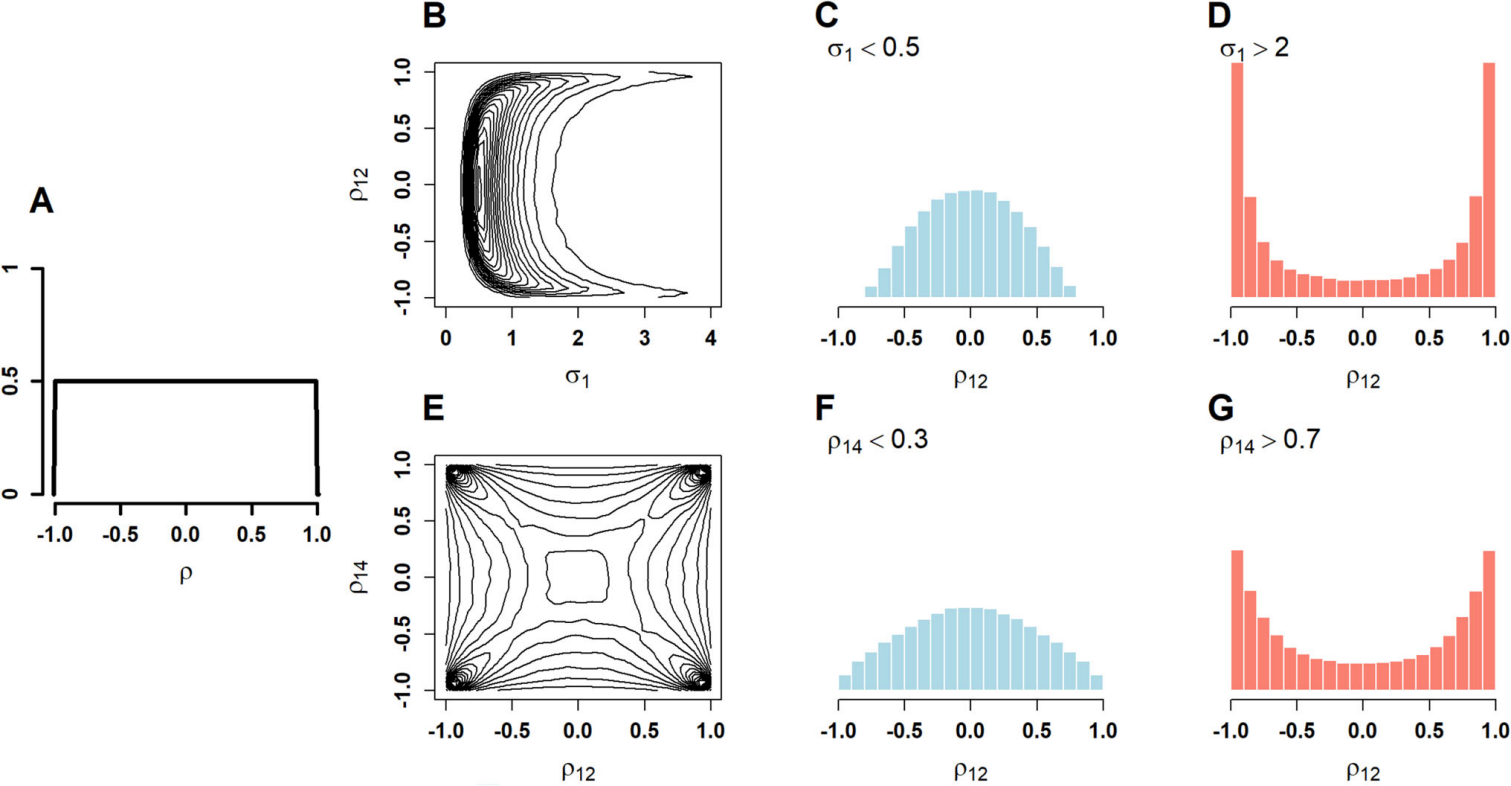
Visualization of the inverse Wishart prior distribution. **A** Marginal prior on correlation (pairwise correlation coefficients). **B** Joint prior on standard deviation and correlation. **C, D** Priors on correlation conditioned on low and high variability, respectively. **E** Joint prior on two correlation coefficients. **F, G** Prior on one correlation coefficient conditional on low and high values of another, respectively

In Bayesian analysis, priors are needed on parameters. In the manifest model, priors are needed for mean $\boldsymbol{\mu }$ and variance $\boldsymbol{\Sigma }$. In the hierarchical model, priors are needed for these two as well as for trial variances $\tau _j^2$. In practice, prior on $\boldsymbol{\mu }$ and the collection of trial variances are not problematic as diffuse priors work well. The critical component is the variance matrix $\boldsymbol{\Sigma }$, especially in the hierarchical model. The goal then is to pick a good prior on $\boldsymbol{\Sigma }$ so that the collection of correlations may be faithfully recovered without undue influence.

In the next section, we review some choices of prior for $\boldsymbol{\Sigma }$. Before doing so, we note that in most multivariate studies of performance, researchers estimate correlations en route to further analyses, such as factor modeling or structural equation modeling. So, why study correlations themselves? Bollen (1989) notes that factor models and structural-equation models impose constraints on correlation matrices. In comparison, we are studying *unconstrained* priors—priors that do not restrict the researchers to explore possible patterns without unwarranted assumptions. These models then serve as a general model against which constrained models, that is, specific factor model or structural-equation models, may be compared.

## Priors on covariance and correlation

In this section, we provide three common choices of prior and explore how the constraints within them could affect invariance to scale and invariance to inclusion. We visualize the covariances using insights from Tokuda et al. (2025).

*Inverse Wishart Prior.* The inverse Wishart distribution (IW) is a popular choice for a prior on the covariance matrix $\boldsymbol{\Sigma }$ because it is conjugate for normally distributed observations. When a covariance matrix follows an inverse Wishart, we write:

$$ \boldsymbol{\Sigma } \sim \text {Inverse Wishart}(\boldsymbol{S},v), $$

where $\boldsymbol{S}$ and $v$ are prior settings that must be chosen beforehand. The prior setting $v$ is often referred to as the degrees-of-freedom of the distribution. It may be set to $v=J+1$ as a default, where $J$ is the size of the covariance matrix (the number of tasks or measures). The more critical setting is that of the scale matrix $\boldsymbol{S}$. This matrix may be diagonal, and the diagonal entries, set beforehand, are the expected variances. The question then is how these settings affect the posterior of correlation.

Figure 2 shows a series of visualizations of the prior for the default $v=J+1$ and $\boldsymbol{S}=\boldsymbol{I}$, the identity matrix. Figure 2A shows the marginal prior on the pairwise correlations, that is, the off-diagonal elements in $\boldsymbol{\rho }$. These are uniformly distributed on the interval $[-1,1]$, and this distribution holds regardless of the number of tasks $J$. Such a prior is highly desirable, as it does not unduly favor any range of correlations. The top row shows how the marginal prior on correlation depends on scale ($\sqrt{\Sigma _{jj}}$). Figure 2B is a contour plot of prior joint distribution of a correlation coefficient and the standard deviation ($\sqrt{\Sigma _{jj}}$). Here we see an issue—there is a notable lack of independence. The two histograms on the top row (Fig. 2C-D) highlight the dependencies. These plots are conditional prior densities on $\rho$. Figure 2C is conditional on small standard deviations, and the overweighting of small-magnitude correlations is seen. Figure 2D is conditional on large standard deviations, and the overweighting of large-magnitude correlations is seen. What do these dependencies mean? They imply that the prior depends on the specification of scale and, moreover, that the analyst needs a reasonable sense of the variation. If the analyst sets the scale $\boldsymbol{S}$ too small, the data are concordant with relatively high variabilities, and posteriors may be biased toward large-magnitude correlations. Conversely, if the analyst sets the scale $\boldsymbol{S}$ too large, the data are concordant with relatively low variabilities, and posteriors may be biased toward low-magnitude correlations.

Figure 2E addresses invariance to inclusion. It shows the relationship between two correlation coefficients, or how the inclusion of one variable affects another. The contour plot reveals a lack of independence. Correlation coefficients tend to be similar in magnitude to each other, and there is less prior mass where one is large in magnitude and the other is small in magnitude. This relationship is visualized more concretely in the conditional prior distributions in Fig. 2F, G. Analysts who include additional variables with large-magnitude correlations may bias posteriors toward larger correlations; those who include additional variables with small-magnitude correlations may bias posteriors in that direction.

The advantage of the inverse Wishart prior is computational speed. The prior is conjugate with respect to the normal, hence conditional posterior distributions are also inverse Wishart with updated parameters. The inverse Wishart prior is convenient to sample from, and MCMC chains run quickly and efficiently (as will be seen). The usual critique is that the scale specification is too informative and excludes small possible variance values (Gelman, 2004; Huang & Wand, 2013). We are less concerned with this aspect here—our focus is squarely on the effect of the estimation of correlation.

**Fig. 3:**
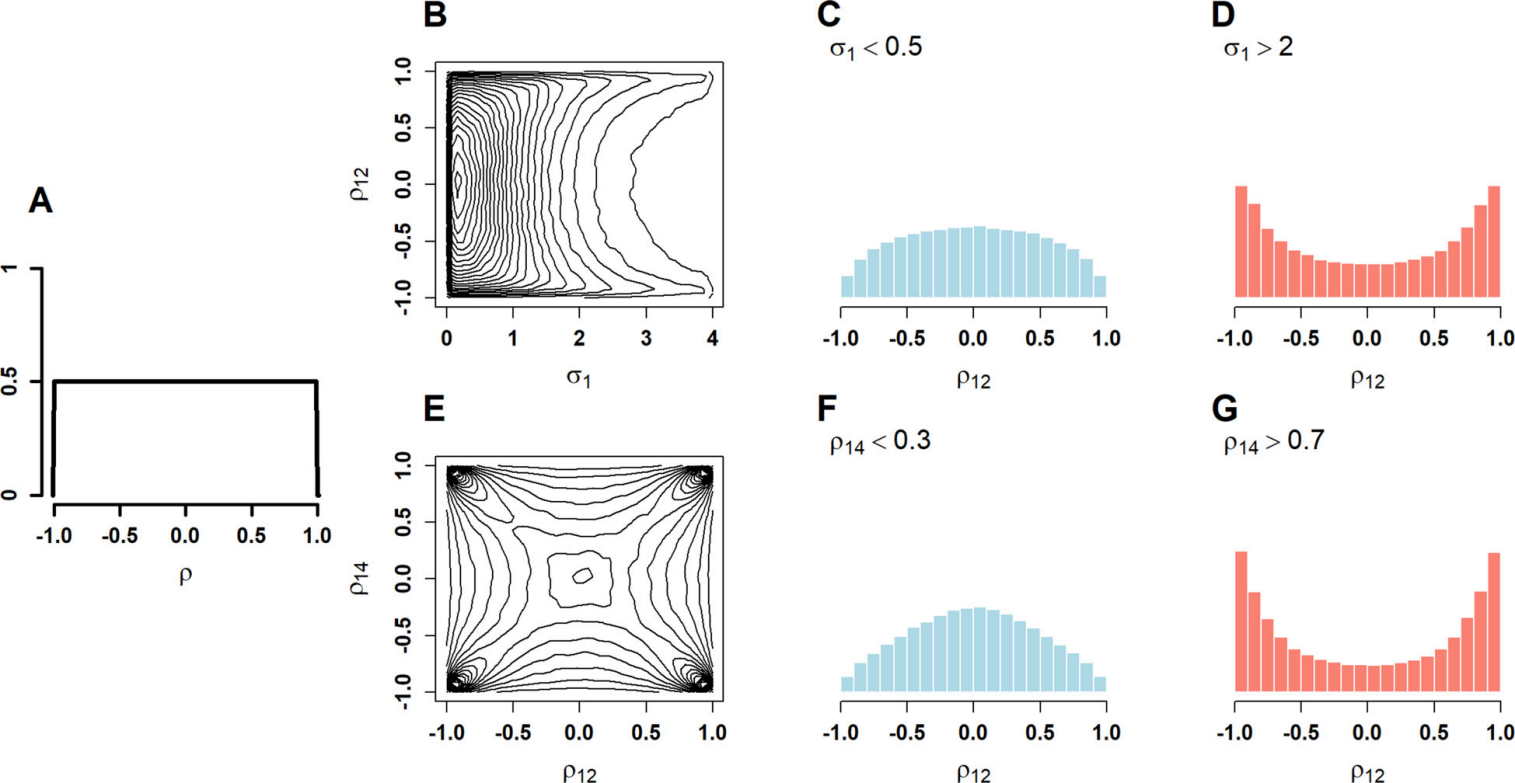
Visualization of the scaled inverse Wishart prior distribution. **A** Marginal prior on correlation (pairwise correlation coefficients). **B** Joint prior on standard deviation and correlation. **C, D** Priors on correlation conditioned on low and high variability, respectively. **E** Joint prior on two correlation coefficients. **F, G** Prior on one correlation coefficient conditional on low and high values of another, respectively

*Scaled inverse Wishart prior.* The scaled inverse Wishart distribution (SIW), from Huang and Wand (2013), is a relatively new prior for covariance. It is a bit misnamed, for it is not a scaled version of the inverse Wishart (which already has a scale setting). Instead, the distribution is formed by taking a continuous weighted mixture of inverse Wishart distributions across all scales. The hope then is that this more diffuse form lessens any burden of specifying a single scale *a priori*. When a covariance matrix follows a scaled inverse Wishart, we write,

$$ \boldsymbol{\Sigma } \sim \mathrm{ SIW }(v,\boldsymbol{S}), $$

where, in this case, $v$ and $\boldsymbol{S}$ are prior settings that must be set beforehand. Prior setting $v$ is referred to as the degrees-of-freedom, though it has a different meaning in the scaled inverse Wishart distribution than in the inverse Wishart distribution. Prior setting $bfs$ is a vector of scales on standard deviations, $\Sigma _{jj}^{1/2}$. The scaled inverse Wishart is related to the inverse Wishart as follows. If $\boldsymbol{\Sigma } \sim \mathrm{ SIW }(v,\boldsymbol{S})$, then,

$$\begin{aligned} &  \boldsymbol{\Sigma } \mid \alpha _1,\ldots ,\alpha _J \sim \text {Inverse Wishart}\!\left( v+J-1,\,2v \,D\!\left( \tfrac{1}{\alpha _1},\ldots ,\tfrac{1}{\alpha _J}\right) \right) , \\ &  \qquad \qquad \qquad \alpha _j \overset{\text {ind}}{\sim } \text {Inverse-Gamma}\!\left( \tfrac{1}{2},\,\tfrac{1}{s_j^2}\right) , \end{aligned}$$

where $D()$ denotes a diagonal matrix. In the inverse Wishart, $v$ references the number of variables, and $v=J+1$ is a useful default corresponding to a uniform prior on all correlations. In the scaled inverse Wishart, the default value is $v=2$ (see Huang and Wand, 2013). With this setting, the marginal priors on correlation coefficients are distributed uniformly on [-1,1], and this distribution holds for all $J$ (see Fig. 3A). The main question revolves around the role of $\boldsymbol{S}$, which are scale values on the standard. Figure 3B shows the joint distribution of variability and correlation for the scaled inverse Wishart prior for comparable settings to the inverse Wishart. Here, there remain some dependencies, though to a lesser degree than with the inverse Wishart. Figure 3C, D shows the conditional prior distributions, and the presence of the dependence, though to a lesser degree may be seen. Figure 3E,G shows, respectively, the joint and conditional distributions of correlation coefficients. The behavior here is the same as the inverse Wishart—the prior tends to make correlation coefficients similar in magnitude and hence violate, at least to some degree invariance to inclusion.

**Fig. 4:**
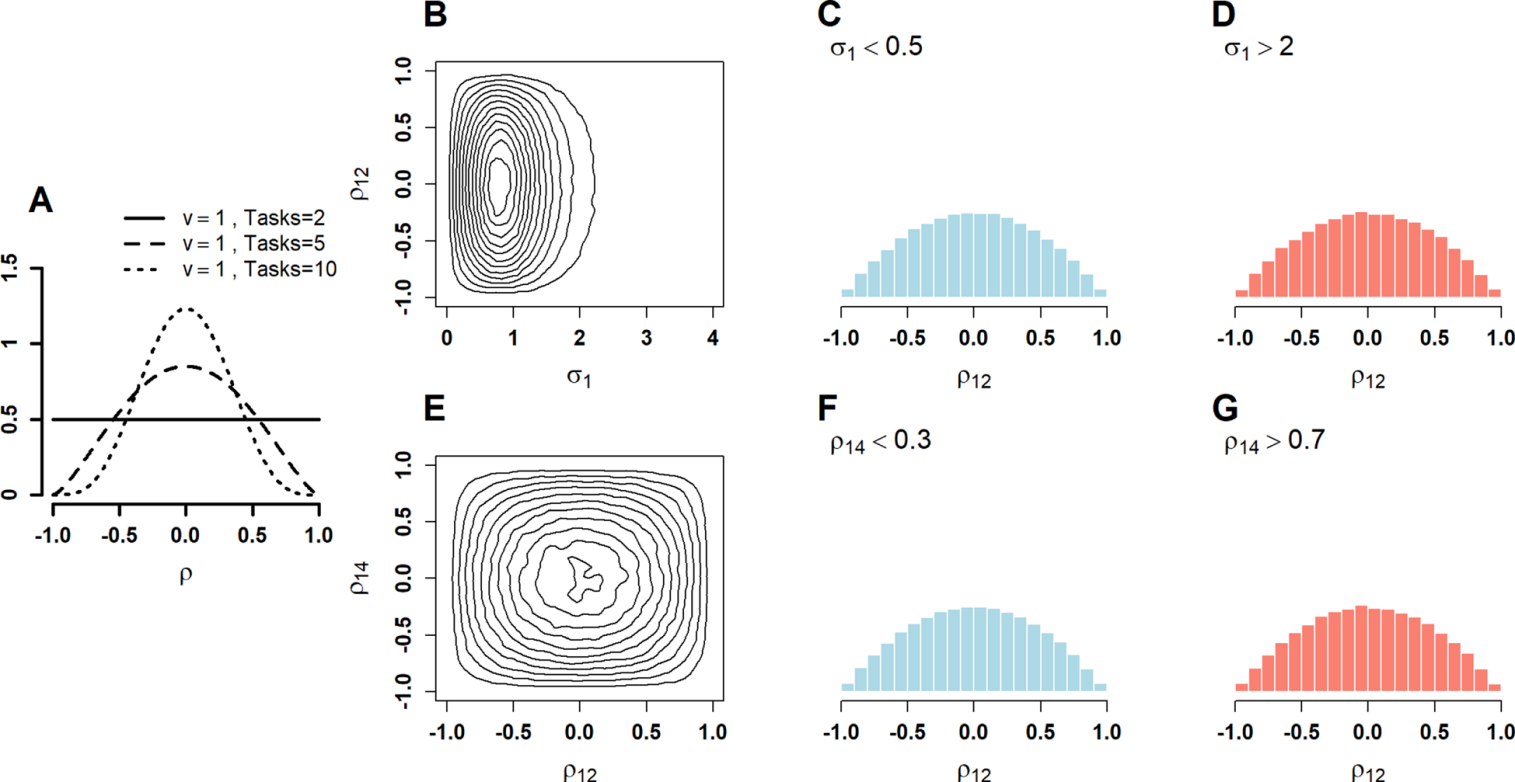
Visualization of the LKJ distribution. **A** Marginal prior on correlation (pairwise correlation coefficients). **B** Joint prior on standard deviation and correlation. **C, D** Priors on correlation conditioned on low and high variability, respectively. **E** Joint prior on two correlation coefficients. **F, G** Prior on one correlation coefficient conditional on low and high values of another, respectively

*LKJ Prior.* The LKJ prior (Lewandowski et al., 2009) is known as a least-informative prior. This prior makes use of Equation (1)—rather than placing priors directly on $\boldsymbol{\Sigma }$, priors are placed on $\boldsymbol{\Sigma }$, the vector of standard deviations, and $\boldsymbol{\rho }$, the correlation matrix. These specifications are separate and decouple variance and correlation.

$$ \begin{aligned} \sigma _j&\sim \text{ Half-t }(2,s_j),\quad j = 1, \ldots , J,\\ \boldsymbol{\rho }&\sim \text {LKJ}(v), \end{aligned} $$

The distribution labeled “Half-t” is the positive half of a scaled $t$ distribution with 2 degrees of freedom and scale $s_j$. The vector $\boldsymbol{S}=(s_1,\ldots ,s_J)$ serves as scale settings that must be set before analysis, and plays a similar role as that in the scaled inverse Wishart. The LKJ prior is a distribution specifically for correlation matrices, and the distribution depends on the shape parameter $v$. The default setting is $v=1$, and the marginal distribution on the correlation coefficients is shown in Fig. 4A. Unlike the inverse Wishart and scaled inverse Wishart, this distribution depends on the number of variables or tasks, $J$. When the distribution is bivariate, $J=2$, the prior distribution on correlation is uniformly distributed on [-1,1]. However, as more tasks are added, the prior emphasizes smaller-magnitude correlations, which is a violation of invariance to inclusion.

Figure 4B–D shows the relationship between variability and correlation, and there is no relationship by construction. This independence is visualized in the conditional histograms (Fig. 4C, D). Likewise, there is no relationship between different pairwise correlations (Fig. 4C, D). The LKJ violates invariance to inclusion by downweighting smaller values with more variables; the inverse Wishart and scaled inverse Wishart priors do so by making correlation values more similar to each other in magnitude.

The LKJ prior is noninformative in the sense that with $v=1$, all possible correlation matrices are equally likely. This uniformity is different than placing a uniform on a single correlation element within the matrix. The LKJ prior on any correlation element does change because the positive-semi-definite property becomes more salient with an increasing number of tasks—it is the positive-semi-definite property that rules out matrices with large magnitude elements (Martin, 2020). The question then for researchers is do they wish uniformity on elements (inverse Wishart, scaled inverse Wishart) or uniformity on correlation matrices taken as a whole (LKJ). Those preferring the latter will gladly accept prior dependence of each element on the number of tasks as it is a natural part of the preference.

## Effect of prior specification for the manifest model

The discussed priors are broad defaults that are used repeatedly throughout the literature. We expected that in the manifest case with little trial noise, they would each be excellent choices. To explore the effects of prior specification on the measurement of correlation, we used the anthropometric data from the U.S. Army (Army, 2012). In the data set, over 6000 soldiers provided about 100 different body measures. We examined the 4082 identified male soldiers, and correlated the height with weight for a random sample of $I=200$ of them. To set the scale, we used the standard deviation of the full sample of 4082 as a reference. For example, the standard deviation of height was 6.86 cm, and we used this number as a baseline. To assess robustness to misspecification, we took this value and manipulated it from 1/10th of the baseline to ten times the baseline, or in this case, from 0.69 cm to 68.6 cm. This is a huge range of variation for an empirical scientist. We estimated either the height or the weight alone as a bivariate problem, or included another eight anthropometric measurements as a ten-variable problem.

**Table 1:** Estimates of correlation under Pearson, IW, SIW, and LKJ priors across different scale multipliers

		Exclude		Include	
Scale	Method	Mean	95% CI	Mean	95% CI
0.1x	Pearson	0.50	[0.38, 0.59]	0.50	[0.38, 0.59]
	IW	0.50	[0.39, 0.59]	0.50	[0.39, 0.60]
	SIW	0.49	[0.39, 0.60]	0.50	[0.39, 0.59]
	LKJ	0.49	[0.38, 0.59]	0.45	[0.35, 0.54]
1x	Pearson	0.50	[0.38, 0.59]	0.50	[0.38, 0.59]
	IW	0.49	[0.38, 0.59]	0.50	[0.39, 0.59]
	SIW	0.49	[0.38, 0.59]	0.50	[0.39, 0.59]
	LKJ	0.49	[0.38, 0.59]	0.45	[0.36, 0.54]
10x	Pearson	0.50	[0.38, 0.59]	0.50	[0.38, 0.59]
	IW	0.47	[0.36, 0.58]	0.48	[0.36, 0.58]
	SIW	0.49	[0.38, 0.59]	0.50	[0.39, 0.59]
	LKJ	0.49	[0.38, 0.60]	0.46	[0.35, 0.55]

Table 1 shows the effect of prior for both the bivariate case (“Exclude” for exclude the other variables) or the ten-variate case (“Include” for include the other variables) for the range of scales. The entries show the point estimate and CIs of the correlation between height and weight. The Pearson sample correlation, as shown, does not vary for include and exclude cases. Overall, results from priors are fairly similar and match the sample-correlation results well. There are two relatively minor deviations. First, posterior estimates from the inverse Wishart prior are a tad too low when the standard deviation scale is ten times the baseline, and this underestimation reflects the prior dependencies shown in Fig. 2B–D. Second, the posterior estimates from the LKJ are a tad too low when additional variables are included. This underestimation reflects the dependence on the marginal prior on correlation with increasing numbers of variables, as shown in Fig. 4A.

The above application shows that all three prior classes do well in the manifest case. There is sufficient prior mass in all cases that data with as few as 200 cases are more than sufficient to dominate the prior. It matters little here which of the three priors is used. Stated alternatively, there is little reason to use Bayesian analysis at all for the manifest case; the sample correlation, along with Fisher’s *z*-transform confidence intervals, provides the same summary.

**Fig. 5:**
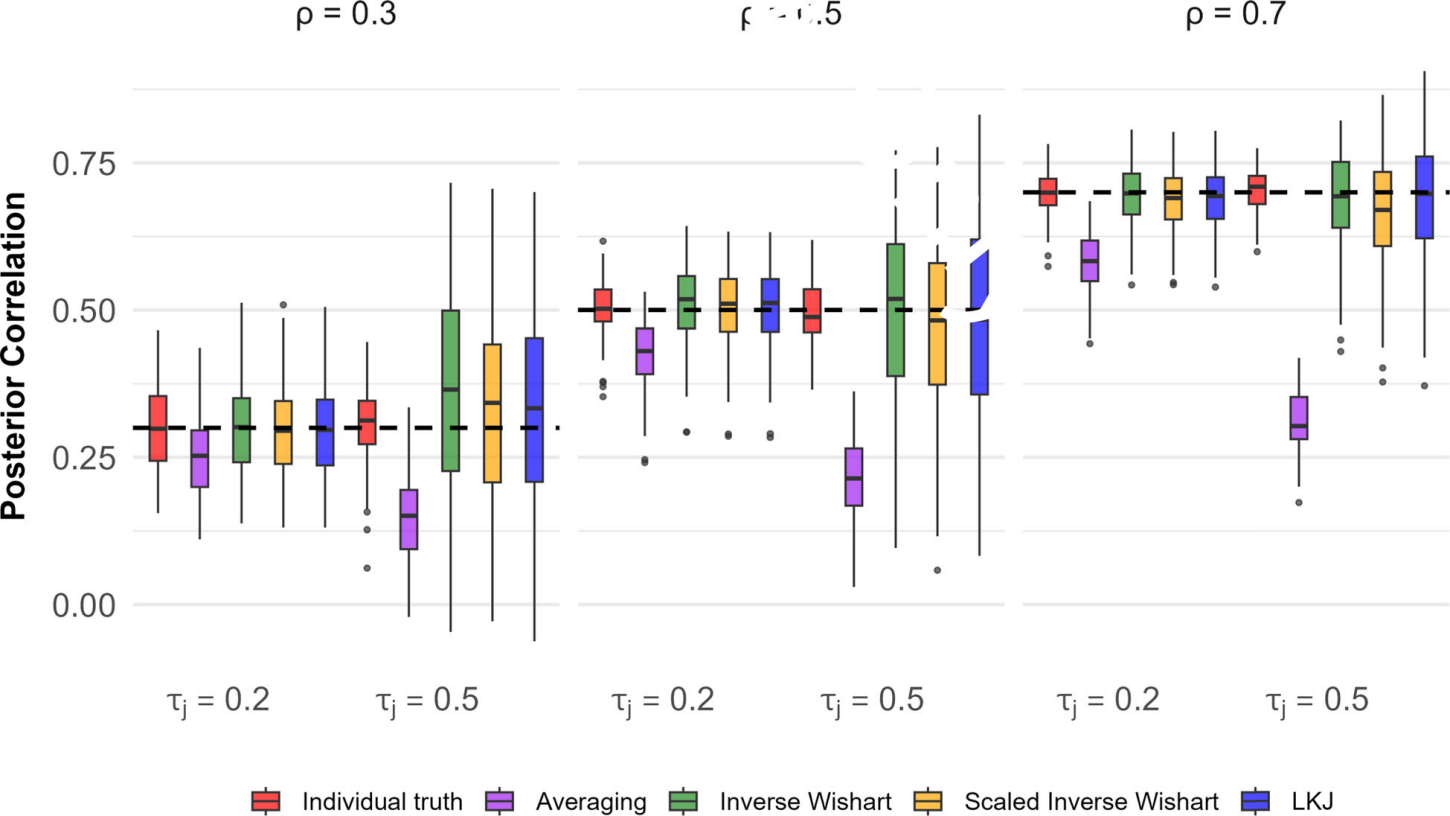
The recovery of correlation for two tasks. There is noticeable attenuation from averaging across trials. There are only slight differences among the three prior specifications

The manifest case is only appropriate when there is little measurement noise. Tolerably low measurement noise, such as in weight measures, is the exception and not the rule in experimental psychology. In fact, in all cases we know of, there is a high degree of measurement noise. And in all these cases, the Pearson correlation coefficient is inadvisable due to substantial attenuation. Hence, the critical question is how these priors perform when the data contain measurement noise, which is modeled in a hierarchical context.

## Simulation study 1: Two tasks. Calibrated scale settings

Simulations in this paper follow a common form. First, to generate data, true individual parameters ($\boldsymbol{\theta }_i$) are sampled from a multivariate normal parent distribution. For two tasks, each individual has two true values with one for each task, and there is a single true population correlation parameter $\rho$. The value of $\rho$ is set as ground truth, and the estimate of which serves as the target of analysis. Here are the settings for the parent distribution on $\boldsymbol{\theta }_i$:

$$ \boldsymbol{\theta }_i \sim \text{ N}_2\left( \begin{pmatrix} .5\\ .5\end{pmatrix}, \begin{pmatrix} .1^2 & \rho \times .1^2\\ \rho \times .1^2 & .1^2\end{pmatrix} \right) , $$

In all the simulations reported here, there were 200 synthetic individuals, that is, $I=200$. Trial level data were sampled from true individual values $\boldsymbol{\theta }_i$ as $Y_{ij\ell } \sim \text{ N }(\theta _{ij},\tau _j^2)$. To speed simulations and draw sharp contrasts between estimation approaches, we set a low number of trials, $L=20$, throughout. True correlations were set to $\rho =.3$, $\rho =.5$, and $\rho =.7$ to cover a variety of cases; trial variance $\tau ^2_j$ was set either to a low-noise value of $.2^2$ or to a high-noise value of $.5^2$, and these values held for all tasks.

In each simulation, trial-level data were generated from the above steps and analyzed with hierarchical models that varied only in the prior specification on $\boldsymbol{\Sigma }$. One specification was the inverse Wishart prior with shape set to the default setting of $v=3$ and scale $s^2$ is set to $.1^2$, which matches the true between-individual variation. Another was the scaled inverse Wishart prior with default shape, $v=2$, and scale $s=.1$, which matches the true between-individual standard deviation. The final specification is the LKJ with prior shape the default of $v=1$ and prior scale $s=.1$. The inverse Wishart and scaled inverse Wishart prior specifications do not have an explicit parameter $\rho$, but it is straightforward to compute a value of $\rho$ on each iteration of the MCMC chain using Eq. (1). The LKJ has lower-triangle matrix outputs on each iteration; the cross product of this matrix yields the posterior correlation matrix.

**Table 2:** RMSE with calibrated priors for individual variation

		$\tau _j = 0.2$			$\tau _j = 0.5$		
Case	True correlation	IW	SIW	LKJ	IW	SIW	LKJ
2 Tasks							
	0.3	0.043	0.043	0.042	0.168	0.153	0.154
	0.5	0.042	0.041	0.041	0.162	0.164	0.17
	0.7	0.034	0.036	0.036	0.079	0.103	0.105
4 Tasks							
	0.3	0.047	0.044	0.048	0.192	0.148	0.151
	0.5	0.045	0.047	0.047	0.119	0.116	0.121
	0.7	0.037	0.048	0.044	0.081	0.121	0.107

**Fig. 6:**
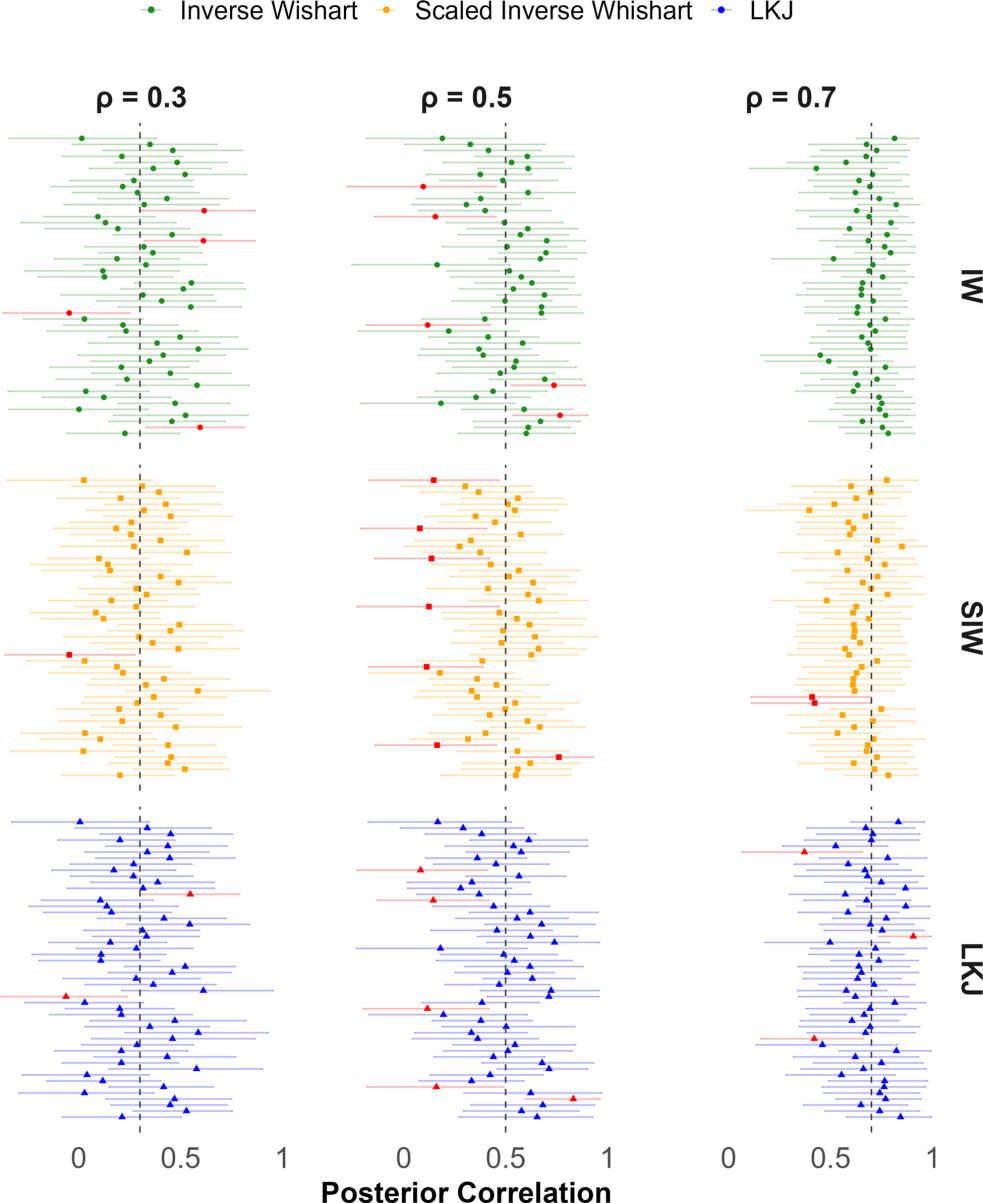
Means and 95 credible intervals for recovered correlation from three prior models across the first 50 runs. Intervals that fail to cover the true correlation are colored in red. These occur at an expected proportion for all prior specifications

**Fig. 7:**
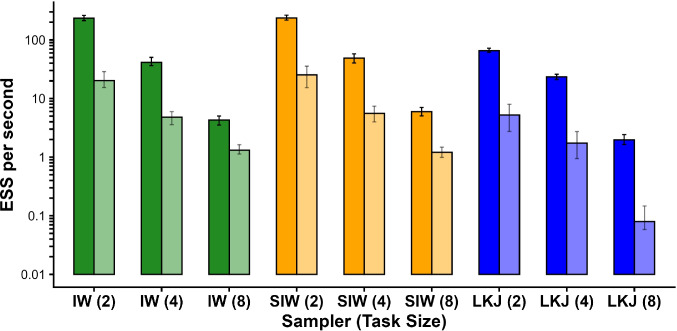
Effective sample size (ESS) per second for three prior models in the two-task, four-task, and eight-task cases for low and high noise. ESS was computed using the bulk ESS measure in (Vehtari et al., 2021). Darker and lighter bars correspond to low noise and high noise cases, respectively

A simulation consisted of 100 replicate runs. New data were generated on each replicate, and the same data were submitted to analysis by each model. The inverse Wishart model and the scaled inverse Wishart model were implemented in JAGS (Denwood, 2016; Plummer, 2003) using a Gibbs sampling algorithm. For each model, 1000 burn-in iterations were followed by 3000 iterations retained for posterior computation. Because JAGS does not support the LKJ prior, the LKJ model was implemented in RStan (Carpenter et al., 2017) using the NUTS algorithm. Again, there were 1000 burn-in iterations followed by 3000 retained iterations. Mixing was assessed through the Gelman–Rubin potential scale reduction factors (R-hat, Brooks & Gelman, 1998). Throughout this report, R-hat was less than 1.01 and 1.10 for all simulations with low noise ($\sigma ^2=.2^2$) and high noise ($\sigma ^2=.5^2$), respectively.

The results of the simulation are shown in Fig. 5. Consider the five left-most boxplots colored in red, purple, green, yellow, and blue. The true correlation here is $\rho =.3$, as indicated above and as denoted by the dashed line. For these five boxplots, there is a low degree of trial noise as indicated by $\tau _j=.2$. The red boxplot is the distribution of sample correlations among the 200 individual true values across the 100 runs. The variation serves as what is expected from finite samples of 200 people if there were an infinite number of trials. We use this as a best-case outcome as individual true-values are known. The remaining four boxplots are based on the trial-level data. The purple boxplot is the distribution of correlations from the averaging method, where averages are tabulated across $L=20$ trials. As expected, this distribution is slightly biased low. This bias is the attenuation of the correlation due to measurement noise. The bias is not that severe because there is not a high degree of trial noise. The green, yellow, and blue boxplots show the distributions of the posterior means of the correlation parameter across runs for the three different priors. These are similar to the correlations among true values, indicating that the hierarchical model with any of the three priors does an excellent job of accounting for trial noise. The results for the high-trial-noise case follow in the next four bars with the subtitle $\tau _j=.5$. The following trends hold: (i) there is much attenuation for averaging (purple boxplot); (ii) correlation recovery is somewhat more variable for the hierarchical models; and (iii) the performance does not vary appreciably across different priors. The remaining panels show the results with higher true correlations. The results are largely the same, except that attenuation from averaging becomes more pronounced as the true correlation increases. Overall, there is little to distinguish the performance of inverse Wishart, scaled inverse Wishart, and LKJ priors in these simulations.

One way to characterize the results is to compute the RMSE for each estimate across the 100 simulation runs. Table 2 shows these RMSE values, and the pattern is clear. For low noise and true correlation of .3, the hierarchical model with any of the three priors is about as accurate as knowing the individuals’ true values. There is a loss in the high-noise case, but the performance is about the same.

One of the most useful benefits of Bayesian hierarchical analysis is that posterior distributions themselves provide estimates of how well parameters are estimated. The relevant quantity is the *posterior credible interval* (Kruschke, 2014), which is somewhat analogous to a confidence interval (cf., Morey et al., 2016). The credible intervals for the first 50 runs in the high-noise case are shown in Fig. 6. Consider the upper left-hand plot, which is for a true population correlation of $\rho =.3$. There are 50 horizontal lines for the 50 runs. In this case, each line is a posterior credible interval for the correlation coefficient for the run, and the dot is the posterior mean. For $\rho = 0.3$ and $\rho = 0.7$, for all three priors, the vast majority (96%) of credible intervals contain the true value. For $\rho = 0.5$, this proportion was slightly lower (88%) but still reasonably high. Overall, the credible intervals appear to provide satisfactory coverage. The simulations show encouraging results with all three priors.

One of the primary advantages of the inverse Wishart and scaled inverse Wishart priors is their computational convenience. The inverse Wishart is conjugate to the normal distribution, which makes sampling relatively inexpensive. This computational efficiency enables the faster execution: In Simulation 1, LKJ prior’s sampling efficiency was several times lower than inverse Wishart’s and scaled inverse Wishart prior’s (see Fig. 7). This multiple in efficiency grows with the variability in the data and with the size of the covariance matrix.

**Fig. 8:**
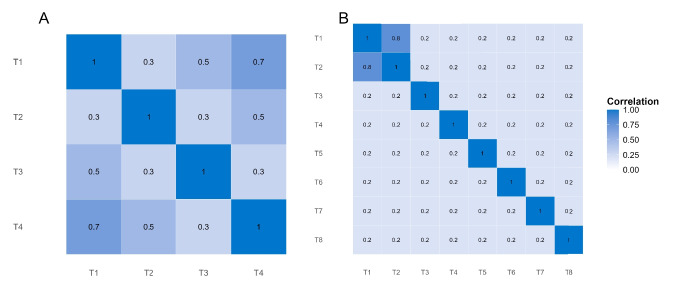
Ground truth correlations. **A** Simulation 2 with four tasks. **B**. Simulation 4 with eight tasks

**Fig. 9:**
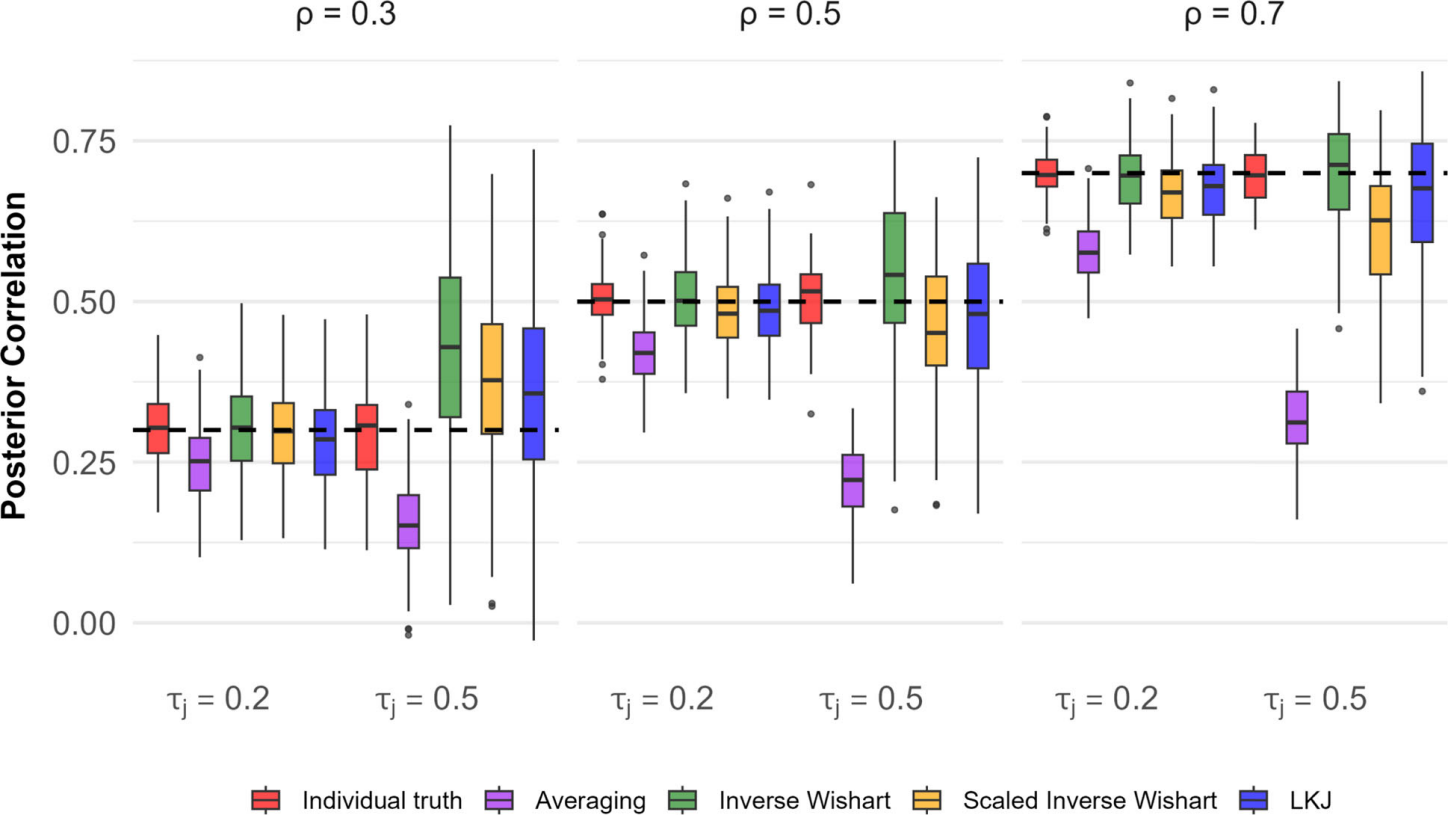
The recovery of correlation for four tasks. There is substantial attenuation from averaging across trials. There are moderate deviations from true values for all prior specifications across the 100 runs, except that under high trial noise and low true correlation, the inverse Wishart prior tended to over-cover the correlation

**Fig. 10:**
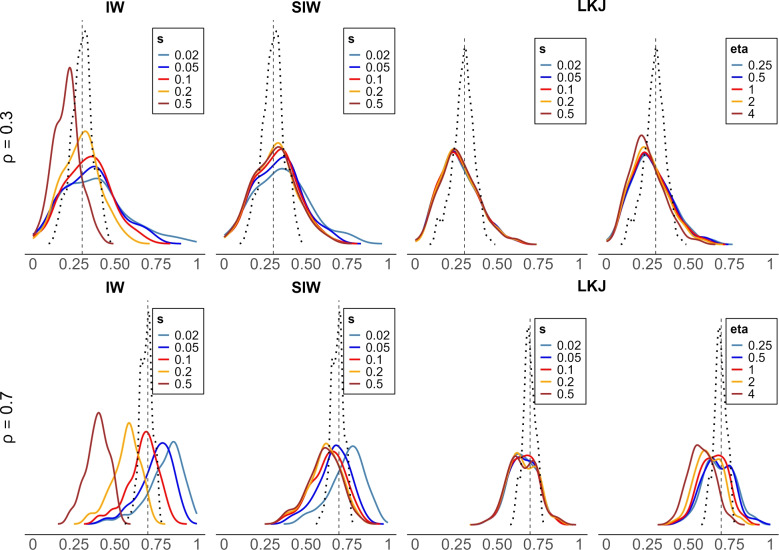
Posterior correlation estimates for four tasks under three models with varying prior settings. Each density curve represents the posterior correlation distributions across 100 runs per condition. The dotted curve shows the individual truth, while the vertical dashed line denotes the true correlation. Posterior means of correlations are highly sensitive to prior scale settings for the inverse Wishart prior, and less sensitive to prior scale settings for the scaled inverse Wishart prior. In contrast, posterior means are much more stable to this prior variation with the LKJ prior. Posterior means are also stable in the LKJ prior with respect to variation in the prior shape parameter. These stabilities make the LKJ prior an attractive choice when there is little guiding prior knowledge

Although this computational-time gap is substantial in relative terms, it should not be overstated. For a single dataset, analyses with any of these priors typically complete within a few minutes, and the difference is mainly relevant in large-scale simulation studies. We also note that real-time speeds may vary depending on model parameterization. We have used an intuitive and straightforward parameterization. There may be alternative parameterizations that improve sampling efficiency in Hamiltonian-based sampling than the more straightforward one used here. More importantly, the LKJ prior offers critical advantages in robustness, which motivates our recommendation to consider it for analyzing real-world data.

## Simulation study 2: Four tasks with calibrated priors

The results in Simulation Study 1 show the benefits of hierarchical modeling and the relative equivalence of the three priors. The study, however, covered just two tasks and a single correlation coefficient. We performed Simulation Study 2 with four tasks. The four-task simulations follow almost all the same settings in Simulation Study 1 with the exception of the ground truth correlation matrices. The true correlations for the four-task versions are shown in Fig. 8A. The four-task true correlation matrix followed a two-factor pattern with set values of $\rho _{12} = 0.3, \rho _{13} = 0.5, \rho _{14} = 0.7$.

**Table 3:** RMSE without calibrated priors for individual variation

		s				
True correlation	Prior	0.02	0.05	0.10	0.20	0.50
0.3						
	IW	0.186	0.147	0.119	0.094	0.115
	SIW	0.152	0.121	0.108	0.105	0.105
	LKJ	0.117	0.115	0.116	0.115	0.116
0.5						
	IW	0.186	0.138	0.105	0.103	0.206
	SIW	0.139	0.108	0.103	0.105	0.105
	LKJ	0.104	0.101	0.101	0.101	0.1
0.7						
	IW	0.153	0.109	0.087	0.144	0.304
	SIW	0.113	0.093	0.109	0.117	0.121
	LKJ	0.09	0.091	0.089	0.092	0.094

We ran the same simulation study with these new correlation matrices and assessed how well correlations may be recovered with the averaging method and three priors. The results for the four-task setup are shown in Fig. 9 and Table 2. These results are presented in the same format as those for Simulation 1, and the results are quite similar. As before, correlations from trial averaging have noticeable attenuation; there is much improvement with the hierarchical models, and performance is similar with three priors.

## Simulation study 3: Robustness to scale settings

Simulation Studies 1 and 2 are best-case scenarios in that the prior setting on variability matched the variance used in data generation. We cannot be so lucky in the application because we do not know the true scale. As discussed previously, the inverse Wishart exhibits dependency between correlation and variability, meaning that poor settings of scale may bias the posterior of the correlation coefficients. And the scaled inverse Wishart has the same dependency, though not to as large a degree. We saw only a minor effect of these posterior settings for the manifest model, as the data for $I=200$ observations were sufficient in number to dominate the prior. Restated, all three priors demonstrate robustness to scale setting in that context. Yet, hierarchical models are more complex and more heavily parameterized. Consequently, the influence of the prior is often much greater.

How does the setting of the scale of variance, $s^2$ affect posterior estimation of correlation in a hierarchical model with measurement noise? This question is addressed by Simulation Study 3 where $s$ (or $s^2$) is manipulated across a few levels. To match the data-generation process in the previous simulations, we set $s=.1$ (or $s^2=.01$). Here, we take values that are either double or half this value, as well as values that are five times or 1/5th this value, that is $s=.02,.05,.1,.2,.5$. We reran Simulation Study 2 with four tasks and with intermediate trial noise of $\tau _j^2=.4^2$.

Figure 10 shows sets of smoothed densities. The densities are *not* posterior densities of the correlation coefficient. They are the distribution of the posterior mean across the 100 replicates. Consider the dotted line in the left-most upper panel. This panel is for the inverse Wishart for true correlations of .3. That dotted line is the sample correlation among true individual values, and it is not perturbed by trial noise. It corresponds to the red boxplots in Figs. 5 and 9, and it serves as a best case. The solid lines correspond to different settings of $s$ for the inverse Wishart. As can be seen, there is much dependence on $s$. This behavior is not desirable. The lower row shows the same for true correlations of .7, and the trend here is even more obvious. The second column shows plots for the scaled inverse Wishart prior; it is less sensitive to prior settings compared with inverse Wishart. However, when $s$ is 5 times smaller than the true value, it has a tendency to overestimate the correlation.

The third and fourth columns show similar plots for the LKJ prior. The third column is for changes in $s$, and the behavior here may be directly compared to that for the inverse Wishart prior. Here, we see the desired robustness of posterior correlation to prior variance scale settings. Table 3 shows the RMSE values for these comparable prior settings—they favor the LKJ when the setting is far from the data-generating value. Because the LKJ fared favorably, we also explored the robustness to settings of $v$, the prior setting on correlations. The values of $v$ were manipulated through $v=.25,.5,1,2,4$, and the results for true low correlation values exhibit the same robustness. The only scenario where there was an effect of prior settings is for higher true correlations, as shown in the lower panel on the right.

## Simulation study 4: Robustness to inclusion

None of the three priors is invariant to inclusion. For the inverse Wishart and scaled inverse Wishart priors, there is increased prior density for correlation coefficients that are similar in magnitude (see Figs. 2E, G and 3E, G); for the LKJ prior there is decreasing density for extreme magnitude correlations as the number of variables is increased (see Fig. 4A). Do these dependencies affect posterior estimates in reasonably-sized data?

**Fig. 11:**
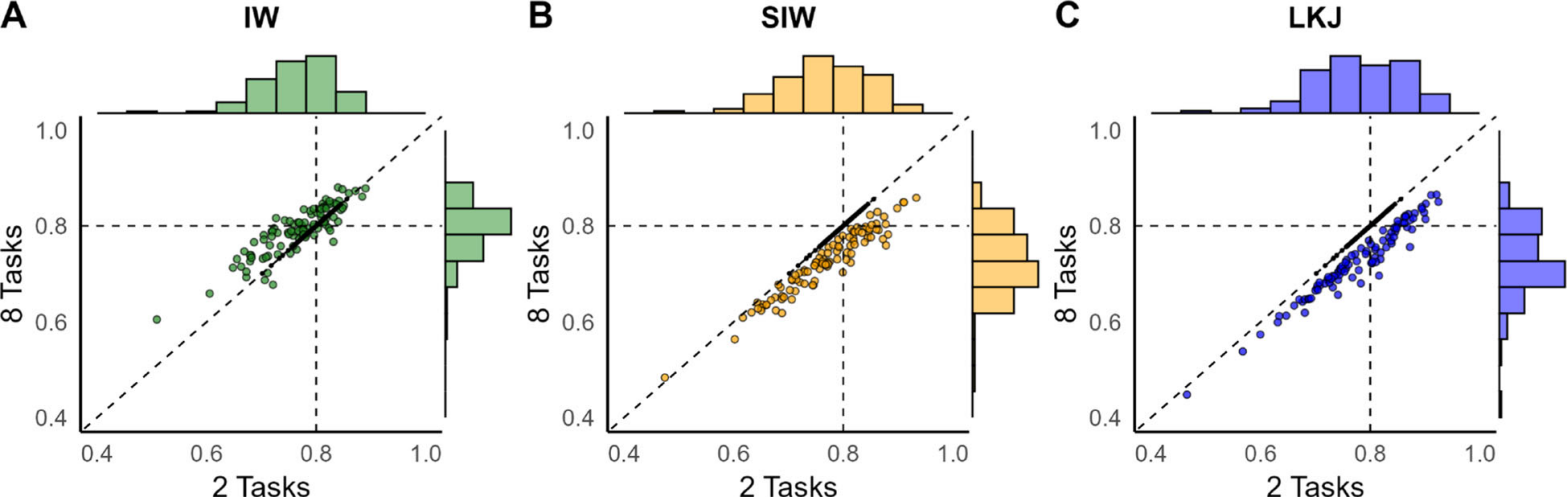
Robustness to inclusion of additional variables. The scatter plots show the relationship of posterior correlation estimates, whether in isolation (*x*-axis) or in the context of eight tasks (textity-axis). There is some downward influence from the prior with inclusion for the scaled inverse Wishart prior and LKJ priors

To explore the issue, we constructed ground truths with correlations shown in Fig. 8B. The key correlation is that between Tasks 1 and 2, which was set to the high value of .8. In one case, the data from Tasks 1 and 2 were submitted to a bivariate analysis, much as in Simulation 1. In a second case, data from all eight tasks were submitted to an 8-dimensional multivariate normal model, and the focus was on the estimate in the correlation between Task 1 and 2 with the six other tasks included and simultaneously analyzed. The trial noise was moderate at $\tau =.4$ and prior scales on variability were set to. $1\hat{\phantom{0}}2$ (inverse Wishart prior) or .1 (scaled inverse Wishart and LKJ priors) as in Simulation 1.

Figure 11 shows the results as scatter plots. First, focus on the small black dots on the diagonal. These are the correlations between true individual values for Tasks 1 and Task 2, and these are the same whether two tasks or eight tasks are analyzed as sample correlation is invariant to inclusion. There are 100 of these points corresponding to the 100 simulation runs. The colored larger points are posterior mean estimates for the correlation between Task 1 and Task 2. The *x*-axis is for the two tasks alone in a bivariate analysis; the *y*-axis is the same correlation when the other six tasks are included. For the inverse Wishart, the estimates are well-centered whether the two tasks were considered in isolation or in the eight-task context. For the SIW and LKJ, the prior dependency is more clear. There is a tendency toward lower posterior means overall, and this effect is accentuated in the eight-task case as expected. As an aside, we had expected similar behavior for the inverse Wishart and scaled inverse Wishart, so we reran the inverse Wishart simulation with different seeds to check. We obtained the same patterns. We are not sure why the inverse Wishart fared so well, but are reasonably confident this finding is not a statistical fluke.

RMSE values for Simulation 4 are reported in Table 4. One critical comparison is between the estimates of .8 true-valued correlation coefficient for the two-task vs. eight-task context. The inverse Wishart prior is slightly better in the eight-task case than the two-task case, and this result was replicated on an additional simulation with different seeds. The RMSE is slightly worse in the eight-task case than the two-task case for the SIW and LKJ, and given the systematic pattern of underestimation in Fig. 11, this result is more interpretable. Table 4 shows that while the estimate of the high correlation value between Task and Task 2 was accurate for the inverse Wishart, the estimate of the low correlations between the other tasks was less accurate. This accuracy reflects an inflation of the low correlation values for this prior.

## Simulation study 5: Robustness to distribution of variability for LKJ prior

In the inverse Wishart and scaled inverse Wishart priors, the analyst places a prior on the covariance that encompasses both the variance and correlation. The resulting marginal prior on variance is inverse gamma for the inverse Wishart prior, and the marginal prior on standard deviation is a half-$t$ for the scaled inverse Wishart. The LKJ prior is qualitatively different in that separate priors are placed on variability and correlation. The analyst is free to choose any form for the prior on the standard deviation they wish. We chose a half-$t$ distribution. Does this choice matter?

**Fig. 12:**
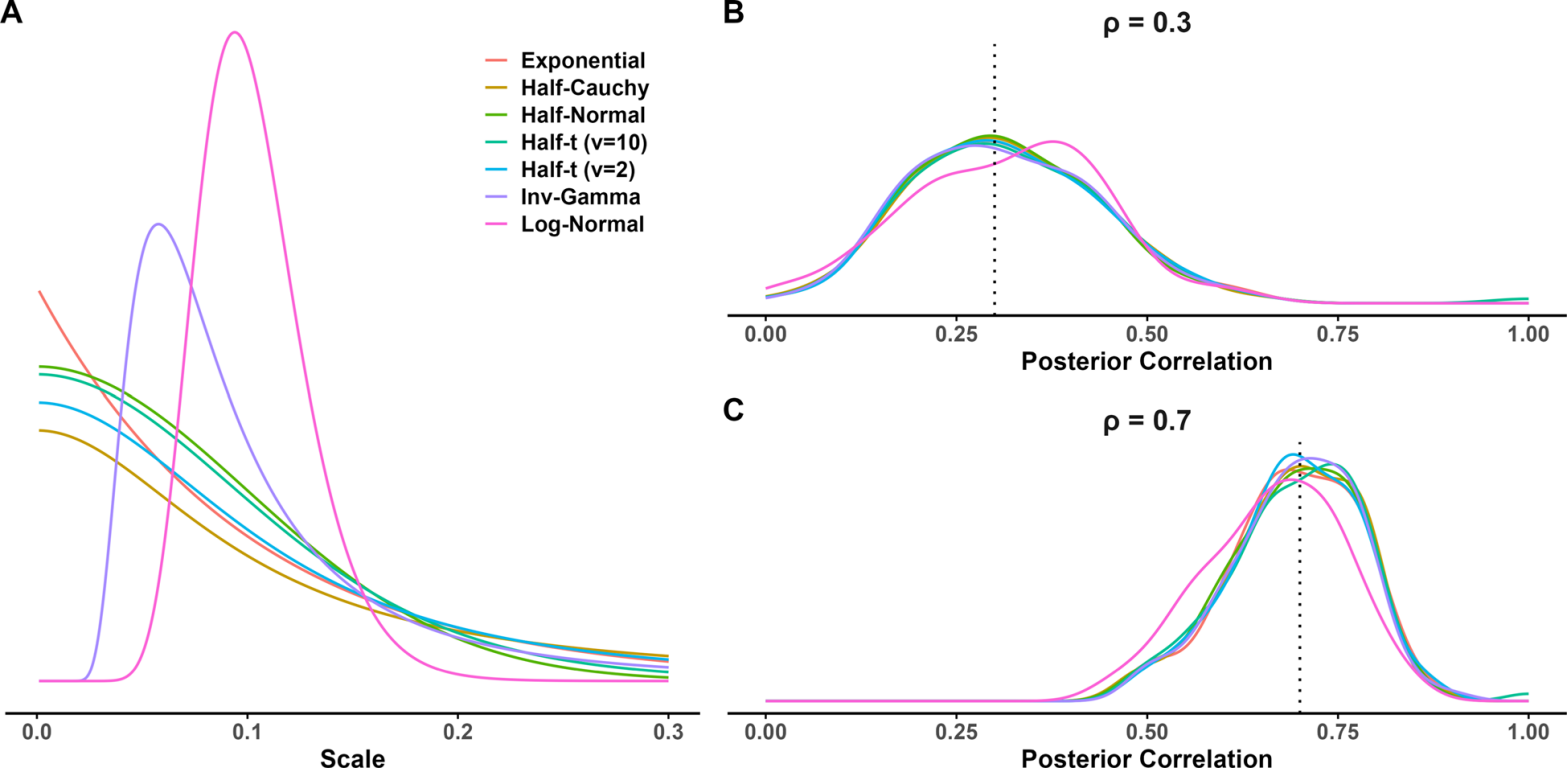
Different scale prior distributions’ impact on correlation estimates in LKJ model. **A** Seven selected scale prior distributions. All of them were set to center around the true scale value of 0.1. **B** The posterior correlation estimates for low and **C** high correlation. All scale prior distributions resulted in similar posterior correlation estimates

One reason to suspect this choice does not matter is that there is prior independence between the variability and correlation. Hence, any dependency comes from the likelihood itself. Nonetheless, to explore the possibility, we ran a brief simulation with seven different distributions on standard deviation in the LKJ prior. The distributions are shown in Fig. 12A, and the center of these distributions was at a value around $s=.1$.^1^ Although the priors differ in shape and tail behavior, they all share a common reference point—the center—allowing a reasonable comparison of their influence on the posterior correlation distribution.

**Table 4:** RMSE in eight-task inclusion case

Case	True correlation	IW	SIW	LKJ
2 Tasks				
	0.8	0.064	0.073	0.08
8 Tasks				
	0.2	0.157	0.137	0.102
	0.8	0.056	0.093	0.097

The simulation setup was the four-task setup in Simulation 2, with the correlations shown in Fig. 8A, which has correlation values of .3, .5, and .7. The upper right panel shows the distribution of posterior means as a smoothed density for the 100 replicates for each prior. The true value here is .3. As can be seen, the choice of prior form matters little, perhaps with the exception of the lognormal distribution. The lower plot shows the case for the true value of .7, and the same result holds. Overall, it seems to matter little which broad distribution is used for the standard deviation in the LKJ prior so long as it has broad coverage and tails at least as fat as an exponential.

## Simulation 6: Contrasts

The preceding simulations are designed to provide insight into the performance of the three priors and to be computationally convenient. For these purposes, we used simplified models that do not account for experimental conditions and contrasts. The generalization of the hierarchical model setup is straightforward. Consider, for simplicity, tasks comprised of two contrasting conditions. Examples include Stroop or flanker tasks (congruent vs. incongruent), priming tasks (primed vs. not primed), or task-switching (repeated vs. switched). The key in all these tasks is that the measure of interest is a contrast, such as the difference between incongruent and congruent conditions or between repeated and switched conditions. The following model centers this contrast. An observation is denoted $Y_{ijk\ell }$ where $i$ denotes the individual, $j$ denotes the task, $k=1,2$ denotes the condition, and $\ell$ denotes the replicate trial. The data model is

$$ Y_{ijk\ell } \sim \text{ N }(\alpha _{ij}+x_k\theta _{ij},\tau _j^2), $$

where $\alpha _{ij}$ is the overall mean or intercept for the $i$th person in the $j$th task, $x_k=-.5,.5$ is the contrast code for the $k$th condition, $\theta _{ij}$ is the contrast or slope for the $i$th person in the $j$th task, and $\tau _{j}^2$ is the variability across replicate trials in the $j$th task. The target of interest is $\theta _{ij}$, the contrast, and the previous individual latent multivariate normal model, $\boldsymbol{\theta }_i \sim \mathrm{ N}_J(\boldsymbol{\mu },\boldsymbol{\Sigma })$ is appropriate. Also, as before, priors are needed on $\boldsymbol{\Sigma }$.

The main difficulty with the inverse Wishart prior is that the prior choice of scale on variability affects the posterior distribution of correlations (see Fig. 10). This problem is less salient for contrast parameters because researchers often have appropriate *a priori* information. Here, we argue that researchers often know at least a rough approximation of the variability of contrast parameters across individuals. The argument starts with one observation about the direction of individual effects: Rouder and Haaf (2021) argues that most experimental manipulations yield individual true contrasts that are only in one direction. Take, for example, the Stroop effect, where color naming is quicker on average for congruent than incongruent stimuli. The argument is that each individual has a true Stroop effect in the same direction, and that no one truly names the color of incongruent items faster than that of congruent items. When all individuals have true effects in the same direction, Haaf and Rouder (2017) call it an *everybody does* situation. This *everyone does* situation places substantial limits on the scale of variability. Suppose a contrast has a mean of 60 ms, which is typical of Stroop, flanker, and priming effects. The variability in this effect could not be too large, else a sizable proportion of individuals would have true effects in the opposing direction. For example, if the mean effect was 60 ms, the standard deviation may not be larger than 30 ms, as any more would imply more than 2.5% of the population have the opposite effect. Hence, just by knowing the mean effect, we have good *a priori* information about the variability across individuals. The question is whether posterior distributions of correlations vary appreciably with this knowledge in the inverse Wishart setup.

**Fig. 13:**
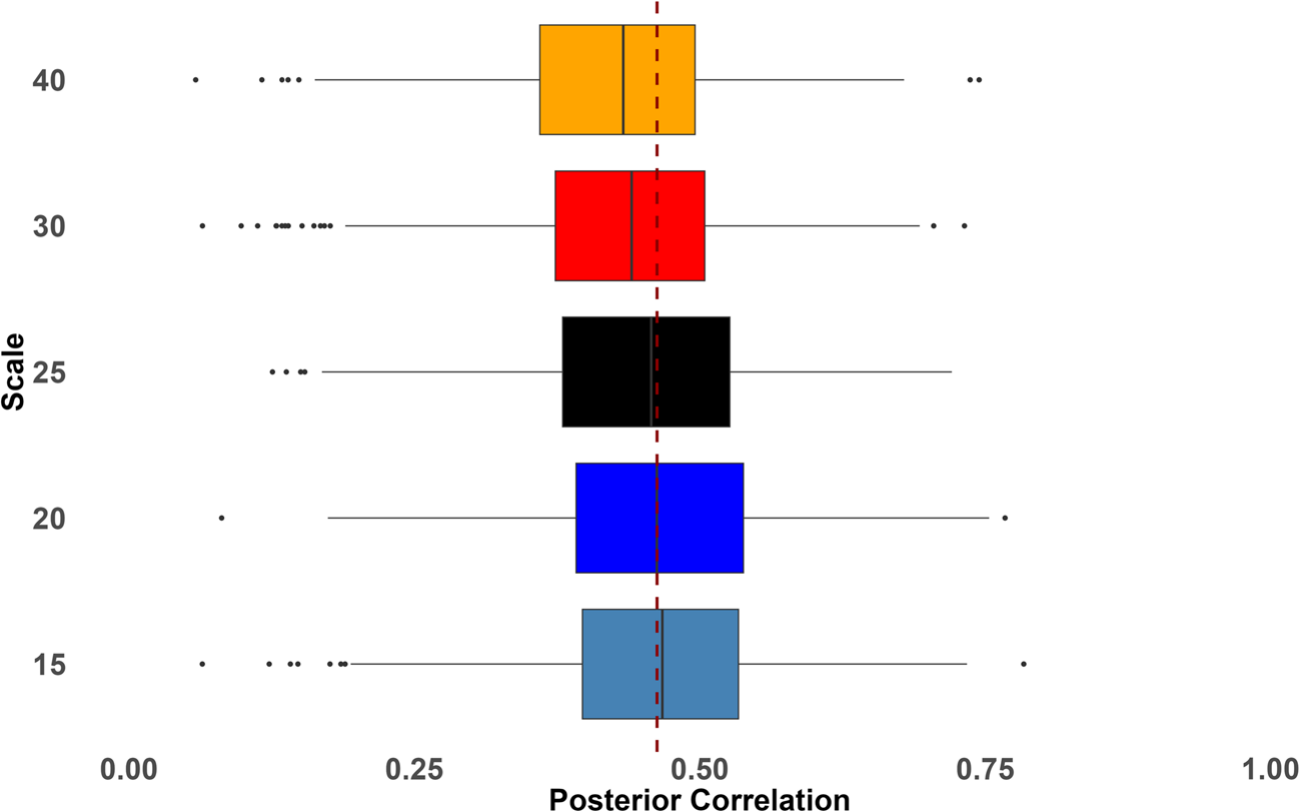
Posterior distributions of the correlation coefficient as a function of prior variance setting. Boxplots do not vary appreciably, showing the stability of these posteriors across a reasonable range of settings. The true population correlation is 0.5; the true correlation among these 200 participants is slightly less and is shown by the *dashed vertical line*

To answer this question, we ran a simulated Stroop experiment with two conditions in each of two tasks—say a color Stroop task and a numerical Stroop task (MacLeod, 1991). The conditions of the simulations were designed to be typical of several Stroop data sets we have examined (see Haaf et al., 2024). As before, there were 200 synthetic individuals. Each ran 150 trials in each task and each condition. Individuals varied in their true overall speed: $\alpha _{ij} {\mathop {\sim }\limits ^{iid}} \text{ Normal }(600,100^2)$. They also varied in their true Stroop effect as follows: These Stroop effects were drawn from a bivariate normal with a mean vector of 60 ms in each task. The standard deviation in each was 25 ms, and the correlation across the two tasks was .5. trial noise, $\tau _j$ was to 175 ms.

To test how the prior choice of scale on variability affects the posterior distribution of correlations, we reran the analysis with several choices of $s^2$. Our guiding principle was that $s^2$ should reflect a range of reasonable prior opinion but no more. Here, for a 60-ms mean, we thought a value of $(40 \text{ ms})^2$ was very large as it implies 7% of individuals truly have a reverse Stroop effect where they truly respond quicker to incongruent than congruent items. Likewise, a value of $(15 \text{ ms})^2$, the mean being four times the standard deviation, strikes us as very small. Hence, the question is how varying values of $s^2$ in this range affect posterior correlation estimates. The resulting posterior correlation distributions are plotted as boxplots in Fig. 13, and the effect of prior scale across this reasonable range is modest at best. In conclusion, the variability issue is less salient for contrast parameters especially when researchers have a good idea what the mean effect may be.

## Discussion

Hierarchical models provide a superior approach to estimating correlations over sample correlations computed from sample means. These models treat trial noise and individual variation separately. As a result, correlation estimates are disattenuated, and uncertainty reflects both variation across individuals and trials. Sample correlations from sample means, in contrast, have neither of these sanguine properties—they are dramatically attenuated and the associated confidence intervals are too small as they do not reflect trial variability. *We recommend that hierarchical models always be used in estimating correlations from experimental data, especially where multiple trials may be used to estimate measurement error.* Analyses without these models run a substantial risk of misinterpretation.

The question then is what prior specification is best for estimating correlation coefficients? In this paper, we have compared the inverse Wishart, scaled inverse Wishart, and LKJ prior specifications in manifest and hierarchical contexts. The recommendation is that the LKJ prior is better for general use with empirical data sets. The reason for this recommendation is that the LKJ prior is less sensitive to prior settings than the others. Our recommendation reflects a visualization of the priors as well as simulations of robustness.

A comparison of the visualization of the priors (Figs. 2-4) shows that the inverse Wishart and scaled inverse Wishart priors have at least some sensitivity to prior scale setting. Furthermore, each is sensitive to the inclusion of additional variables, as correlation coefficients are shrunk toward more similar magnitudes. The LKJ prior on correlation is robust to variance-scale settings, but the marginal prior on correlation becomes more peaked at small-magnitude values as more variables are included in the analysis.

The performance of the priors in simulation reflects in part biases evident in the visualizations of the priors. The dependence in the inverse Wishart of correlation on prior scale manifests itself in simulation—we draw readers’ attention to the difficult results for the inverse Wishart models in Fig. 10. Although the LKJ prior is a safer choice, it is important not to overstate the costs. The scale of variance, the critical prior setting, is not particularly mysterious or unknown. For many applications, especially those with contrasts, the range of reasonable prior scale may indeed be small.

One limitation with the inverse Wishart and scaled inverse Wishart models that is not transparent in our simulations is when the units of measure differ wildly on scale. It would be desirable in practice to have a scale-invariance property. For example, suppose we measured response times in two conditions, and in one we used milliseconds in one and seconds in another. Ideally, the choice, though different, should not matter. It does in the inverse Wishart and scaled inverse Wishart models because of the prior cross dependence on scales. Researchers who have incommensurate scales may wish to scale dependent measures to z-scores if using inverse Wishart or scaled inverse Wishart priors. One way of doing this scaling is to place priors on effect sizes which is implicit in the $g$-prior setup of Zellner (1986).

The choice of the LKJ prior is perhaps the safest. There are two considerations analysts should be aware of when making this choice: the first is that the LKJ marginal prior depends on the number of variables included in an analysis. This makes the choice of how many variables to include salient. Including more variables lowers in magnitude the posterior estimate of each, though the degree of this biasing effect is not overwhelming. The second is that the LKJ prior is computationally slow and does not scale well to higher-dimensional models. For example, for eight variables, high noise cases, the inverse Wishart and scaled inverse Wishart models ran at about one sample per second; the LKJ prior ran at 0.05 samples per second. That factor of 20 may be the difference between 1 day and 20 days of run time. Moreover, this factor increases with increasing numbers of variables, so it is entirely possible to wait weeks and months for an LKJ prior analysis. If the computational demands of the LKJ are difficult to meet, we recommend the scaled inverse Wishart prior as a default alternative to the inverse Wishart. The scaled inverse Wishart prior is far less sensitive to prior scale while providing for just as rapid computations.

Lastly, we remind readers that simulation results are just that. We have simulated a small sliver of possible cases that reflect our best judgment of the community’s needs. Perhaps the thorniest remaining issues involve when to include variables in analyses. We sense that there is more work to be done in understanding the statistical consequences of such choices.

## Data Availability

No human data are collected in conjunction with this manuscript.

## Footnotes

1 Note that some distributions do not have means.

## Code Availability

JAGS and Stan code for the models may be found at https://osf.io/qe9ts.
